# Evaluation of dogs with genetic hyperuricosuria and urate urolithiasis consuming a purine restricted diet: a pilot study

**DOI:** 10.1186/s12917-017-0958-y

**Published:** 2017-02-08

**Authors:** Jodi L. Westropp, Jennifer A. Larsen, Eric G. Johnson, Dannika Bannasch, Andrea J. Fascetti, Vincent Biourge, Yann Queau

**Affiliations:** 1 Departments of Veterinary Medicine and Epidemiology, Davis, CA USA; 2 Molecular Biosciences, Davis, CA USA; 3 Department of Veterinary Surgery and Radiology, Davis, CA USA; 4 Population Health and Reproduction, Davis, CA USA; 5 UC Davis School of Veterinary Medicine, One Shields Avenue, Davis, CA 95616 USA; 6 Royal Canin Research & Development Center, Aimargues, France

**Keywords:** Canine, Urate, Urolithiasis, Diet

## Abstract

**Background:**

Urate urolithiasis is a common problem in breed homozygous for the mutation that results in hyperuricosuria. Low purine diets have been recommended to reduce purine intake in these dogs.

**Methods:**

A higher protein, purine restricted diet with water added was evaluated in dogs with genetic hyperuricosuria and a history of clinical urate urolithiasis over a one year time period. Dogs were evaluated at baseline and 2, 6, and 12 months after initiating the test diet. Bloodwork, urinalysis, abdominal ultrasound, body composition, and 24-h urinary purine metabolite analyses were performed.

**Results:**

Transient, mild, self-limited lower urinary tract signs were noted in only one dog on a single day, despite variable but usually mild and occasionally moderate amounts of echogenic bladder stones (<2-3 mm in size) in almost every dog at each visit. No significant differences were noted in urine specific gravity, urine pH, lean body condition score or body composition. Urinary uric acid concentration was lower on the test diet (*p* = 0.008), but 24-h uric acid excretions were similar (*p* = 0.220) compared to baseline. Significant differences between least squares mean plasma amino acid concentrations measured at the 0 and 12-month visits were found only for valine (*p* = 0.0119) and leucine (*p* = 0.0017).

**Conclusion:**

This study suggests the use of a low purine, higher protein diet with added water may be beneficial as part of the management of dogs with genetic hyperuricosuria and history of clinical urate urolithiasis.

**Electronic supplementary material:**

The online version of this article (doi:10.1186/s12917-017-0958-y) contains supplementary material, which is available to authorized users.

## Background

Urate-containing uroliths comprise approximately 25% of the canine uroliths submitted to the G.V. Ling Urinary Stone Analysis Laboratory [1]. Other data show that 6.4% of canine uroliths were classified as purines when at least 70% of the stone consisted of purine mineral. [2] A gene mutation in the SLC2A9 transporter has been identified as the underlying defect in Dalmatians and other unrelated breeds; this results in hyperuricosuria, a risk factor for urate urolithiasis [3].

Prevention strategies suggested for the management of urate uroliths in dogs with genetic hyperuricosuria include low purine diets (often achieved by feeding a low protein diet), urine alkalinisation, xanthine oxidase inhibitors, and increased water intake [4]. One method of decreasing purine intake is by restricting dietary protein, which can be associated with losses in lean body mass if intake of essential amino acids is inadequate [5]. As such, the amino acid adequacy of purine-restricted, low protein diets might be a concern if the amino acid profile and protein digestibility are not sufficient.

A higher protein but still purine-restricted veterinary therapeutic diet for the prevention of urate urolithiasis in dogs is available [6]. The aim of this study was to evaluate a group of hyperuricosuric dogs with a history of clinical urate urolithiasis that was recommended to consume a purine restricted diet. Our main hypothesis was that dogs consuming the test diet with water added to maintain the baseline urine specific gravity over a 1- year time period would: 1) have lower or similar 24- h urinary uric acid excretion values compared to when consuming their baseline diet. Furthermore, we hypothesized that dogs would 2) improve or maintain body condition and lean body mass, and 3) maintain normal plasma amino acid concentrations. Ultrasonographic examinations of the dogs were also obtained at designated time points to assess for the presence or absence of urolithiasis during the study period.

## Methods

Dogs between the ages of 1–10 years, with a history of confirmed urate urolithiasis with no other comorbidity and 2 copies of the SLC2A9 mutation (confirmed by submitting DNA test; https://www.vgl.ucdavis.edu/) were eligible for enrollment. Exclusion criteria included other systemic disorders that required medical therapy, obstructive ureteral calculi or nephroliths, and medications other than heartworm and flea preventatives. If allopurinol was being administered, it had to be discontinued at least two weeks prior to enrollment. The University of California, Davis Institutional Animal Care and Use Committee, the School of Veterinary Medicine Clinical Trials Review Board, and the Royal Canin Ethics Committee approved the experimental protocol. The dog’s owners signed a consent form prior enrollment.

The test diet was provided free of charge (Royal Canin Veterinary Diet Urinary U/C Low Purine Dry Dog Food; 50 g protein/1000 kcal; Additional file 1: Table S1). Complete physical examination including body condition score (BCS) [7], abdominal ultrasounds, urinalyses (including urinary pH obtained by dipstick and pH meter), and urine cultures were performed at all visits (0, 2, 6, and 12 months). Body composition, plasma amino acid concentrations, CBC, and a serum biochemical panel were analyzed at 0 and 12 months. 24-h urine collections were analyzed at 0, 2, and 12 months.

For two weeks prior to each visit, owners maintained a food diary to record daily food intake in grams, plus treats. Based on BCS, adjustments in the amount fed were provided (to maintain or encourage ideal body condition; weight loss rates not to exceed 0.5% of body weight per week). A list of purine- restricted [8] treats and their calorie content was provided with instructions not to exceed 10% of the daily energy intake as treats (Additional file 2: Table S2). Owners were encouraged to add water to the test diet (which they had previously been instructed to do when feeding the baseline diet) to promote urine specific gravity (USG) for urolithiasis management (<1.020 based on clinical experience). Owners also completed questionnaires at each visit to assess their dog’s appetite, activity level, body condition (including muscle mass), and coat condition, by using subjective scales from 1 to 5 (Additional file 3: Chart S1).

### 24-h uric acid excretions

Because owners were reluctant to have their dogs catheterized and hospitalized for a 24-h period and some dogs would not eat well in hospital, a modified urine collection procedure was implemented. Owners presented their dog to the veterinarian 24 h prior to their scheduled data collection visit. At that time, the bladder was catheterized using aseptic technique and all urine was removed and discarded. The owners were required to collect all urine the dog voided in a clean container over the next 24 h and immediately add the sample to the refrigerated pooled urine. At 24 (+/− 2) hours, the bladder was catheterized and emptied at the VMTH after imaging studies were performed; this urine was added to the pooled samples and the total amount recorded. An aliquot was submitted to the VMTH Clinical Diagnostic Laboratory for uric acid analysis using a colourimetric assay as previously described [9]. The urine was stored at −80 °C and aliquots were sent on dry ice to the Centre Hospitalo-Universitaire de Rangueil in Toulouse, France for determination of uric acid, allantoin, hypoxanthine, and xanthine concentrations using a capillary electrophoresis (CE) method as previously described [10].

### D_2_0 analysis

Body composition analyses were performed as previously described [11] with the modifications described by Villaverde et al. [12] All samples were frozen at −20 °C until analysis.

### Plasma amino acid analysi

Blood was obtained 4–5 h after a meal and plasma separated for amino acid analysis. Two hundred microliters of plasma was removed, and an equal volume of 6% sulfosalicylic acid (with a norleucine internal standard) was added to precipitate protein in the sample. Samples were maintained at −80 °C until analysis. Complete plasma amino acid analysis (of 24 amino acids) was performed as described elsewhere [13].

### Imaging

Complete abdominal ultrasonographic examination was performed at the 0 and 12 month visits; focal urinary tract examinations were performed at the 2 and 6 month visits. All ultrasonographic exams were performed approximately 24 h after the initial urinary catheterization and prior to the final urinary catheterizations to ensure a moderate size bladder for proper imaging. The following were recorded: 1) presence or absence of mineralisation or nephroliths (including size) present in either kidney, and 2) presence or absence of uroliths or small amount of mineral opacities (“sand”) present in the urinary bladder and proximal urethra. For continuity, cystoliths < 2 mm were noted as “sand” while mineral opacities ≥ 2 mm with acoustic shadowing were considered cystoliths; the size and number were recorded if possible. A board certified radiologist (EGJ) reviewed all kidney and urinary bladder images.

### Statistical analyses

A linear mixed model (mixed procedure of SAS) was used to assess the influence of visit number (fixed effect in 2, 3, or 4 levels) on the following parameters: appetite, activity, coat scores, body weight, BCS, % lean body mass, % fat mass, plasma amino acid concentration, urine pH, volume, USG, and urinary concentration and urinary excretion of individual purine metabolites. The dog was included as a random term, as each dog was its own control. According to the residual distribution of each model, data were ranked or not. Data that were ranked (rank procedure of SAS) for non-normal distribution included appetite, activity, and coat quality scores, and for these, as well as BCS, medians are reported. Normally distributed data are reported as least square means ± standard errors.

When more than 2 visits were involved, a Dunnett post-hoc test was used to compare each visit number to visit 1 (initial visit at 0 month). In addition, for urinary concentration and excretion of individual purine metabolites, contrast method was used to compare visits 2 (at 2 months) and 4 (at 12 months) to visit 1.

To investigate correlation between the uric acid measurements obtained at the two laboratories, a general linear model was used. According to the residuals distribution of this model, the Kendall Tau correlation coefficient was determined. Significance level was set at 0.05 for all tests.

## Results

Nine dogs were enrolled in the study; all were castrated males. Six of the dogs had uroliths removed previously that were comprised of 100% urate, one dog had a scant (1%) amount of struvite present in the outer layer, one dog’s urolith was composed of 100% urate in the core and 80% urate and 20% apatite in the outer layer, and one dog’s urolith was 95% urate with 5% calcium oxalate. Breeds included 7 Dalmatians, 1 American Bulldog, and 1 “Miniature” Dalmatian. Two dogs were lost to follow up after the 2 and 6 -month visit, respectively (both Dalmatians). The median age at enrollment was 5 years (range 2–8 years). During the study, one owner reported their dog was stranguric for one day, which resolved without intervention. All other dogs remained free of any upper or lower urinary tract signs including but not limited to stranguria, hematuria, pollakiuria, urethral obstructions, abdominal pain, decreased appetite and inappropriate urinations during the 12-month study period.

### Dietary history

Individual dog histories are presented in Table 1. On a metabolic body weight basis (kg BW^0.75^) dogs were consuming a median of 101.5 kcal/kg BW^0.75^ (range 68.2–134.4 kcal/kg BW^0.75^) at enrollment. One dog was fed a homemade diet formulated to be restricted in purine content (44.5 g protein/1000 kcal; 3.8 g protein/kg BW^0.75^), which consisted primarily of eggs, cottage cheese, pasta, carrots, and kale. Seven dogs were consuming all or most of their calories from a veterinary therapeutic diet formulated for urate urolithiasis (Hill’s Prescription Diet u/d Canine Non-Struvite Urinary Tract Health Dry and Canned Dog Food; 25–29 g protein/1000 kcal; median 2.4 g protein/kg BW^0.75^). One dog was fed a dry adult maintenance diet (Nutro Natural Choice Sensitive Skin and Stomach Adult Venison Meal and Whole Brown Rice Formula Dry; 4.3 g protein/kg BW^0.75^). One dog ate 43% of his calories as a canned adult maintenance diet (Nature’s Recipe Easy to Digest Chicken, Rice and Barley Recipe Cuts in Gravy Canned; total intake 1.8 g protein/kg BW^0.75^). Six dogs received up to 14% of their energy intake from treats. Four dogs received no treats or less than 10% of energy intake from treats.

**Table 1:** History and baseline dietary information

	History	Diet Prior to Enrollment
Dog 1	VUH for cystic calculi 6 years prior, LUTS but not obstructed at that time	Home-cooked diet formulated by nutritionist
Dog 2	Cystotomy for UO 1 year prior and self-limited LUTS 6 months prior to enrollment	Veterinary therapeutic diet formulated for urate urolithiasis and maintenance diet
Dog 3	Cystotomy 3 months prior to enrollment	Veterinary therapeutic diet formulated for urate urolithiasis
Dog 4	UO & cystotomy 2 year prior; 2^nd^ cystotomy 5 months prior; 3^rd^ cystotomy after baseline	Veterinary therapeutic diet formulated for urate urolithiasis
Dog 5	LUTS, Lithotripsy for uroliths one month prior to enrollment	Maintenance diet
Dog 6	Cystotomy 6 months prior to enrollment; UO	Veterinary therapeutic diet formulated for urate urolithiasis
Dog 7	Cystotomy 2 months prior to enrollment; UO	Veterinary therapeutic diet formulated for urate urolithiasis
Dog 8	Cystotomy 3 months prior to enrollment; UO	Veterinary therapeutic diet formulated for urate urolithiasis
Dog 9	Urethrostomy, cystotomy 2 months prior for UO	Veterinary therapeutic diet formulated for urate urolithiasis

LUTS = Lower urinary tract signs

UO = urethral obstruction

VUH = voiding urohydropropulsion; LUTS = lower urinary tract signs

At the 2, 6, and 12-month visits (*n* = 9, *n* = 8 and *n* = 7 dogs respectively), all owners reported their dogs liked the diet “very much” (median score 1/5 at each visit). Median energy intake of the test diet was 83.7 kcal/kg BW^0.75^ (range 33.3–112.3 kcal/kg BW^0.75^); the contribution from treats is included in these amounts (range 0.5–25% of daily energy intake from treats). Median protein intake from the test diet was 4.1 g/kg BW^0.75^ (range 1.6–5.6 g/kg BW^0.75^).

### Compliance

One owner fed excessive treats (22–25% of energy provided as treats) and used treats not on the recommended list provided. Another owner allowed her dog to consume various other foods including an unknown portion of deer carcass primarily between the 6 and 12 month visits. Data from both of these dogs are included in the analyses. The two dogs that were lost to follow up after the 2 and 6 -month visits, respectively were removed from the study because the primary investigators were unable to contact them despite repeated efforts via phone and email.

### Urinalysis and urine culture results

There was no significant difference in USG during the study period when evaluating single USG measurements. Urinary pH, when analysed by urine dipstrip or pH meter was significantly lower among study visits when evaluating spot urine samples at each study visit. Urine pH evaluated by pH meter from the 24-h pooled urine samples was not significantly different among study visits (Table 2). On visit one, one dog had many struvite crystals present, one dog had rare urate crystalluria and one dog had few amorphous as well as rare urate crystals present on urine sedimentation. Of the nine dogs on the second visit, one of the same dogs had rare urate crystals present again and one new dog had a few amorphous crystals present on urine examination. Rare urate crystals were seen again in the same dog on the third exam as well as rare amorphous crystals in the other dog. Furthermore a few amorphous crystals were seen in another dog at this time. No crystalluria was reported at the fourth visit for any dog evaluated. Dog 4 had a non-clinical bacterial urinary tract infection at 3 visits: *Streptococcus* spp. was cultured at baseline and 2 months, and *Pseudomonas* was isolated at the 12- month visit. Oral antimicrobials based on sensitivity testing were administered for 10 days for each instance. This dog’s data were excluded from the urine pH and USG analyses.

**Table 2:** Urine specific gravity (USG) and urine pH as analysed by dipstrip and pH meter from spot urine samples obtained during each study visit. Urine pH analysed by pH meter from 24 h pooled urine samples are also provided

Variable	Baseline	2 month	6 month	12 month	Visit effect *P value*
USG	1.016 ± 0.003	1.014 ± 0.003	1.016 ± 0.003	1.013 ± 0.003	*0.754*
pH (dipstick)	7.28 ± 0.318	5.94 ± 0.312	6.63 ± 0.331	5.61 ± 0.44	*0.003*
pH (meter)	6.41 ± 0.20	6.07 ± 0.255	6.65 ± 0.256	5.73 ± 0.265	<0.001
pH (meter- 24 h pooled analyses)	6.53 ± 0.197	6.42 ± 0.197		7.01 ± 0.197	0.0702

Data are presented as the least square means ± standard error

Urine pH on spot urine samples were significantly lower when compared to the baseline values

### 24-h urinary concentrations and excretions of purines metabolites

Table 3 depicts purine metabolites analysed by the CE method; only urinary uric acid concentration was lower on the test diet compared to baseline (*p* = 0.008). Urinary uric acid concentrations were significantly lower at visit 2 and at visit 4 compared to visit 1 (366 ± 48.2 vs. 549 ± 48.2; *p* = 0.033 and 356 ± 54.6 vs. 549 ± 48.2 mg/L*; p* = 0.036 respectively).

**Table 3:** Urinary purine metabolite analytes measured from 24-h urine collections analysed by the capillary electrophoresis method

Variable	Baseline (visit 1)	2 month (visit 2)	12 month (visit 4)	*P value*
Urine volume	685 ± 167	846 ± 167	956 ± 178	*0.110*
Urine HX conc.	22.9 ± 3.8	18.0 ± 3.8	22.6 ± 4.4	*0.581*
Urine X conc.	16.4 ± 6.1	13.2 ± 6.1	17.0 ± 6.5	*0.766*
Urine UA conc.	549 ± 48.2	366 ± 48.2	356 ± 54.6	***0.008***
Urine ALL conc.	355 ± 83.8	384 ± 83.8	385 ± 92.2	*0.713*
Urine HX exc.	15.5 ± 2.6	12.4 ± 2.6	13.9 ± 2.9	*0.320*
Urine X exc.	9.7 ± 2.1	6.71 ± 2.1	9.7 ± 2.3	*0.405*
Urine UA exc.	361 ± 60.2	306 ± 60.2	271 ± 66.1	*0.220*
Urine ALL exc.	227 ± 32.3	232 ± 32.3	179 ± 36.5	*0.593*

Metabolites are reported as both concentrations (conc; in mg/L) and excretions (exc; in mg/24 h), and urine volume is expressed in mL/24 h. Data are presented as the least square means ± standard error. P values result from the contrast method comparing baseline visit to visits 2 and 4 together. UA = uric acid, HX = hypoxanthine, X = xanthine, ALL = allantoin

**Fig. 1:**
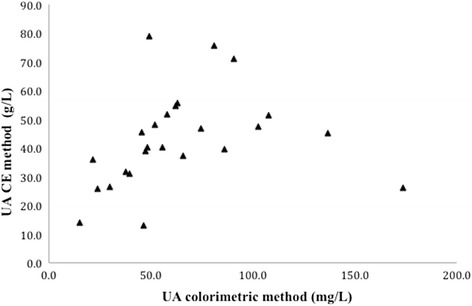
Comparison of the two methodologies (colorimetric and CE) for analyzing urinary uric acid. There was a significant, but weak, correlation when the Kendall Tau test was utilized (*R* ^2^ = 0.52, *p* = 0.0084)

When analysed using the colourimetric assay, no significant differences in uric acid excretions or concentrations were detected among visits (Table 4). Furthermore, there was no significant correlation (*R* ^2^ = 0.06; *p* = 0.24) when comparing the two methodologies. However, there was a significant, but weak, correlation when the Kendall Tau test was used for evaluation (*R* ^2^ = 0.52, *p* = 0.0084; Fig. 1).

**Table 4:** Urinary uric acid measured from 24-h urine collections analysed by the colourimetric assay

Variable	Baseline (visit 1)	2 month (visit 2)	12 month (visit 4)	*P value*
Urine UA conc	669 ± 119	655 ± 119	580 ± 127	*0.573*
Urine UA exc	409 ± 41	401 ± 41	349 ± 45	*0.412*

Data reported as both concentrations (conc; mg/L) and excretions (exc; mg/24 h). P values result from the contrast method comparing baseline visit to visits 2 and 4 together. Data are presented as the least square means ± standard error. UA = uric acid

To assess accuracy regarding the urine collections by the owners, the correlation between urine volume and urine creatinine concentration was assessed with a regression (REG procedure of SAS). The correlation between urine creatinine and urine volume was significant and negative (*p* <0.001, Pearson’s coefficient *R* ^2^ = 0.56; Additional file 4: Figure S1).

### Animal variables and body composition

There were no significant differences noted among visits for the median score for either owner or clinician-assigned BCS, appetite, activity, and coat quality, or for the least square means of body weight, percent lean body mass, and percent fat body mass (Table 5).

**Table 5:** Physical and well-being parameters of dogs consuming the test diet for 12 months

Variable	Baseline (visit 1)	2 month (visit 2)	6 month (visit 3)	12 month (visit 4)	*P value* ^*1*^
BCS_owner^a^	3	3	3	3	*0.603*
BCS_vet^a^	5.5	5.5	5	5	*0.442*
Appetite^b^	1	1	1	1	*0.359*
Activity^b^	2	2.5	3	3	*0.515*
Coat^b^	1	1	1	1	*0.904*
LBM^c^	69.8 ± 3.25	-	-	69.8 ± 3.98	0.991
FBM^c^	30.2 ± 3.25	-	-	30.1 ± 3.98	0.991

^1^P values are from the linear mixed model to assess the effect of visit

^a^Median values for body condition scores (BCS) as reported by the owner and attending clinician. Body condition scores were based on the standard 9 point scale for the clinician’s evaluation, and a 5 point scale for the evaluation by the owner

^b^Median scores for dog’s appetite, activity level and coat condition as perceived by the owner. Score range 1–5, with a lower score representing the best appetite, highest activity level, and coat condition respectively (see Appendix C)

^c^Lean body mass % (LMB) and fat body mass % (FBM) as LS Means ± SE

### Plasma amino acids

Significant differences between least squares mean plasma amino acid concentrations measured at the 0 and 12-month visits were found only for valine (174 vs. 194 nmol/ml; *p* = 0.0119) and leucine (115 vs. 175 nmol/ml; *p* = 0.0017). Least squares mean plasma concentrations of taurine and isoleucine increased at the 12-month visits compared to baseline, but were not significantly different (*p* = 0.0715 and 0.0784, respectively).

### Imaging studies

**Fig. 2:**
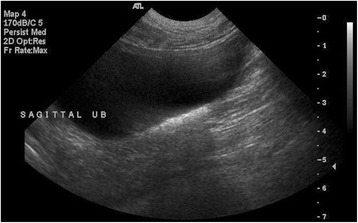
Sagittal ultrasound image of the urinary bladder representative of what was defined as mild sand accumulation

**Fig. 3:**
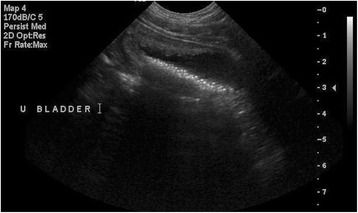
Sagittal ultrasound image of the urinary bladder and is representative of what was defined as moderate sand accumulation (all stones <2 mm)

**Fig. 4:**
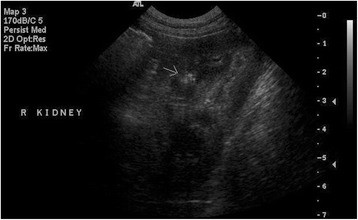
Sagittal ultrasound image of the right kidney and is representative of what was defined as mild renal mineralisation. There is a hyperechoic focus in the renal parenchyma (arrow)

**Fig. 5:**
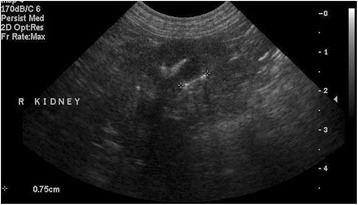
Sagittal ultrasound image of the right kidney and is representative of what was defined as moderate renal mineralisation

**Fig. 6:**
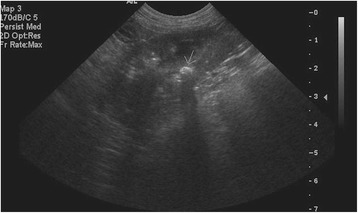
Sagittal ultrasound image of the right kidney and is representative of what was defined as a nephrolith. There is a hyperechoic approximately 5 mm-rounded structure within the renal pelvis, which casts an acoustic shadow indicating mineral (arrow)

Results of the scheduled ultrasound examinations are presented in Table 6. Variable amounts of echogenic sand were noted in the bladder in almost every dog, and were usually mild (Fig. 2) and occasionally moderate (Fig. 3) in severity; however, no lower urinary tract signs were concurrently present. Both renal mineralisation and lower urinary tract sand varied from visit to visit. Renal mineralisation could not be definitively localized to the parenchyma, pelvis, and/or collecting system via ultrasound, but was subjectively evaluated as mild (Fig. 4) or moderate renal mineralisation (Fig. 5), or as definitive nephroliths (Fig. 6).

**Table 6:** Imaging results at baseline, 2, 6 and 12 month visits for the upper and lower urinary tract

	Baseline Imaging	2-month visit	6-month visit	12-month visit
*Upper urinary tract*				
Dog 1	No renal mineralization	static	static	static
Dog 2	Moderate renal mineralization (bilateral)	static	static	static
Dog 3	Moderate renal mineralization (bilateral)	static	static	static
Dog 4	Mild renal mineralization (bilateral)	No renal mineralization	No renal mineralization	Mild left renal mineralization
Dog 5	Mild renal mineralization (bilateral)	static	static	static
Dog 6	Moderate renal mineralization (bilateral)	static	static	Bilateral nephroliths (5.3 and 6.2 mm)
Dog 7	Mild renal mineralization (bilateral)	static	static	static
Dog 8^a^	No renal mineralization in L Mild renal mineralization in R	static	Mild bilateral renal mineralization	N/A
Dog 9^b^	Focal mild renal mineralization (bilateral)	static	N/A	N/A
*Lower urinary tract*				
Dog 1	No mineralization in bladder	static	Mild echogenic sand in bladder	Mild echogenic sand in bladder
Dog 2	No mineralization in bladder	static	static	static
Dog 3	Mild echogenic sand in bladder	static	static	static
Dog 4	Multiple large cystic calculi (largest 7.6 mm)^c^	No mineralization in bladder	Mild echogenic sand in bladder	No mineralization in bladder
Dog 5	Focal pinpoint bladder mineralization	Mild echogenic sand in bladder	Mild echogenic sand in bladder	Mild echogenic sand in bladder
Dog 6	Mild echogenic sand with 4.3 mm cystic calculus	Mild echogenic sand with largest calculi 2.6 mm)	Mild echogenic sand with largest calculi 4.2 mm)	Mild echogenic debris
Dog 7	Mild echogenic sand in bladder	Moderate echogenic sand in bladder	Moderate echogenic sand in bladder with 2.3 mm calculi	moderate echogenic debris with numerous calculi (largest 3.7 mm)
Dog 8^a^	Moderate echogenic sand in bladder	Mild echogenic sand in bladder	Mild echogenic sand in bladder	N/A
Dog 9^b^	Multiple small cystoliths in bladder (largest 3 mm). Suture material present	Mild echogenic sand in bladder	N/A	N/A

^a^lost to follow-up after 6 months

^b^lost to follow-up after 2 months

^c^Surgery done after baseline evaluation

n/a : not applicable

One dog (dog 7) formed numerous cystic calculi between the 6 and 12 month visits; this owner did not adhere to the test diet as described above. The second dog (dog 6) had progressive nephrolithiasis as well as cystic calculi; voiding urohydropropulsion was performed at study completion, and the cystic calculi were analysed as 100% calcium oxalate. This dog’s previous stone composition was 100% urate. Abdominal radiographs were subsequently performed and showed that the renal calculi were strongly radiodense. Radiographs were available for review from the beginning of the study and no evidence of radiodense calculi were noted.

## Discussion

Urinary purine metabolite concentrations in dogs consuming the test diet were similar to baseline values for all dogs that completed the study. The urinary purine values achieved while consuming the test diet may be appropriate for managing these dogs with the SLC2A9 mutation and historical clinical urate urolithiasis. The management of canine urate urolithiasis has involved the restriction of dietary purine often by lowering dietary protein intake in order to decrease concentrations of urinary purine metabolites. In short-term trials in healthy beagle dogs, a casein-based diet formulated with 10.4% protein and 1% potassium citrate (dry matter basis) significantly decreased the urinary activity product ratios of uric acid, sodium urate, and ammonium urate as well as 24-h urinary uric acid excretion compared to a meat based diet with 31.4% protein (dry matter basis) [14]. We found a significant decrease in 24-h urinary uric acid concentrations only at the 6 and 12-month visits compared to baseline when evaluated by the CE method. No other differences were noted with regard to other purine metabolites when evaluated by either method. It is unclear at this time why only one variable was significantly different. It was likely not due to urine dilution because the urine specific gravity was not significantly different over time. Furthermore, no other purine concentrations were significantly lower. These results suggest that the test diet may be suitable alternative for managing dogs with genetic hyperuricosuria and a history of clinical urate urolithiasis.

While studies in dogs are not published regarding the possible side effects of long-term protein restriction, it has been demonstrated that protein requirements increase in older dogs secondary to increased protein turnover [15], and the impact of low protein diets in individuals with lower energy requirements might be more pronounced. We did not find any significant difference at 12 months compared to baseline with regard to body composition or most plasma amino acid concentrations. This was interesting as most dogs in this study were younger or middle aged at enrollment, and their maintenance energy requirements (MER) were relatively low with a median of just 88% (range 35–118%) of the estimated values for inactive pet dogs established by the National Research Council (MER = 95 x kgBW^0.75^) [16]. As such, of the 7/9 dogs eating the lower protein diet at the baseline visit, 4 were not eating protein in concentrations to meet the NRC minimal requirement (2.6 g/kgBW^0.75^), while 2 dogs were consuming protein in amounts that fell between the minimal requirement and the recommended allowance, and only 1 exceeded the recommended allowance (3.3 g/kgBW^0.75^). After consuming the test diet for 2 months, all dogs were ingesting protein in concentrations that exceeded the NRC recommended allowance per metabolic BW. By the 6 and 12 month visits, one dog’s energy requirements had decreased so that in order to maintain stable body weight the amount of test diet was reduced to the extent that the protein intake fell below the NRC minimal requirement. Regardless, we did not find any significant difference at 12 months compared to baseline with regard to body composition or most plasma amino acid concentrations for any dogs. However, our sample population was small and we did not compare essential amino acid profiles among the diets. Larger, longer- term studies may be warranted to examine the benefit, if any, of higher dietary protein concentrations.

We noted significant differences between two different methodologies for analyzing urinary uric acid. The CE method is fast and simple; however, the careful preparation of all standards is necessary for validation. Because this method is not readily available at our institution, we commonly use the colourimetric assay, which is useful for evaluating urinary uric acid trends. The primary clinical indication for determination of 24-h urinary uric acid excretion is to titrate the dosage of allopurinol [9].

Decreasing the urinary concentration of calculogenic substances by increasing urine volume is one of the cornerstones of urolithiasis prevention [17]. In the current study, mean USG was maintained <1.020 at every visit and was not significantly different from baseline, likely because we encouraged owners to continue to provide added water for their dogs. The test diet is only available as a dry formulation, but all dogs consumed the test diet readily with appropriate amounts of water added to produce target USG (<1.020). This test diet with added water appears to maintain low urinary purine metabolite excretion for dogs that require a purine-restricted diet. However, USG should be monitored periodically in dogs with clinical urolithiasis.

Aciduria is considered a risk factor for urate urolithiasis because ammonium and hydrogen ions may precipitate with uric acid [18]. In the current study, urinary pH was lower than historically recommended [18] for urate urolithiasis management at all visits and regardless of methodology. Urinary alkalinizing agents such as potassium citrate could be considered to increase the urine pH, although studies suggest supplementation in healthy dogs may have inconsistent effects on urinary pH [19].

While consuming the test diet with added water (2-, 6- and 12-month visits for the 7 dogs that completed the study and 2- and 6-month visits for 2 dogs lost to follow up), most dogs only had mild echogenic sand in their bladders with the exception of the dog whose owner did not strictly adhere to feeding only the test diet to her dog, which developed cystic calculi at the 6- and 12-month visits. While other studies have not reported the presence or absence of renal mineralisation, we noted these findings were also subjectively static throughout the one- year study period in the dogs that completed the trial. Despite their predisposition due to gender and genetics, all dogs in the current study remained free of upper or lower urinary tract signs, despite variable amounts of mineralization noted periodically. All dogs were managed without medications such as urinary alkalinizing agents or xanthine oxidase inhibitors while consuming the test diet with added water. However, urinary sand was present in 5/7 dogs at study completion; it is unknown if lowering urinary urine acid excretion any further would be of benefit in these dogs. Furthermore, they remained free of any clinical signs, so further intervention was not initiated.

Urate calculi are not often radiodense, and contrast cystourethrograms or ultrasonography is considered more sensitive for detection of uroliths. Radiography was only able to detect 32% of cases with urate uroliths in one study, with many more dogs that required contrast cystourethrograms [20]. We opted to utilize only ultrasonography in this study for our subjective assessment of urinary mineralisation, as sedation is required at our institution to perform contrast imaging and would preclude other tests during the same visit, which required scheduled meal consumption. Actual urolith recurrence rates could not be determined in this study due to varied stages of disease of the dogs at study enrollment. Furthermore, while we did follow the dogs for one year, recurrence rates are variable and could extend past this time point. However, when evaluating clinical signs only 1 dog in our trial exhibited signs suggestive of lower urinary tract disease, and resolved without intervention.

Two dogs did develop uroliths (1 dog with cystoliths; poor diet compliance and one dog with cystoliths and nephroliths determined to be calcium oxalate). However, in the dog that developed calcium oxalate urolithiasis, not all lower urinary tract calculi were removed and none of the nephroliths were removed. The owner did not wish to pursue another surgery, due to lack of clinical signs and the intervention required. The role of diet and any other individual or environmental factors in the formation of calcium oxalate urolithiasis in this case is unknown. The urinary pH was significantly lower at the end of the study period when spot urine samples were evaluated, which could have contributed to calcium oxalate formation in this dog. The dog was monitored and a customised homemade diet was instituted after study completion to manage the complex urolithiasis. Based on this case, if progression occurs in a dog with known genetic hyperuricosuria, radiographs and ultrasound together are warranted in order to aid in identification of potential development of different types of calculi. Urine pH (preferably several spot evaluations or 24-h pooled samples) should also be evaluated to help with management strategies. Further, any subsequent calculi should be removed and submitted for analysis to aid in management, regardless of history. Of the 1650 calculi that have been submitted from Dalmatians to our laboratory, only 3/1650 (0.001%) contained only calcium oxalate, although 43/1650 (0.03%) had some portion of calcium oxalate mixed with urate or another mineral (unpublished data, University of California, Davis Stone Laboratory, Westropp, 2014).

The limitations of the study include the small sample size. It was difficult to acquire additional dogs that could visit our facility at the scheduled time periods over a one year time period, and we only included dogs with a history of clinical urate urolithiasis. We did this to ensure the purine-restricted test diet was clinically indicated for their disease process. Furthermore, actual urolith recurrence could not be accurately evaluated because some of the mineralisation noted could not be removed prior to study enrollment. Furthermore, the dogs were only studied over a one- year time period. Finally, dogs had variable states of disease when enrolled in the study. While small amounts of echogenic “sand” were visible in the bladder in some dogs during the trial, this sediment was not removed and analyzed. We assumed this sediment was comprised of urate; however, infrared spectroscopy would be required to confirm composition. It is also possible that catheterisations performed 24 h prior to the ultrasonographic evaluations could have inadvertently removed small amounts of tiny cystoliths, therefore underestimating the number of cystic calculi present at the time of imaging.

Finally, 24-h urine collections were obtained from samples that owners collected, and the dogs were not housed in metabolism cages nor had indwelling urinary catheters placed. However, there are well known limitations to entire collections of urine even in controlled environments, which impacts the accuracy of nitrogen balance studies.[21] Probably more importantly in the case of veterinary patients, many dogs do not consume their typical intake of food and water under the conditions of stressful confinement in a hospital setting, which is a large factor influencing the accuracy of assessments based on 24-h urine collections regardless of whether catheterization or free catch techniques are used. The significant negative correlation noted between urine creatinine and urine volume suggests owners did not miss a micturition.

## Conclusions

This study evaluated the use of a low purine veterinary therapeutic diet with added water and suggests it may be beneficial as part of the management of dogs with genetic hyperuricosuria and history of clinical urate urolithiasis. Urinary uric acid concentrations were lower, and the concentrations and excretions of all other purine metabolites analysed were not different compared to baseline values despite a higher protein intake. This study suggests that urinary tract mineralisation may be a common yet incidental finding in some dogs with genetic hyperuricosuria.

## Additional Files

Additional file 1: Table S1. Average nutrient analysis of the test diet. (DOCX 68 kb)
Additional file 2: Table S2. List of acceptable low purine treats provided to owners. (DOCX 39 kb)
Additional file 3: Chart S1. Owner questionnaire provided at each visit. (DOCX 215 kb)
Additional file 4: Figure S1. Evaluation of the correlation between urine volumes to urine creatinine. There was a normal distribution of the residuals, as well as homoscedasticity (indicating homogeneity of the variance and the absence of outliers). The correlation was highly significant and negative (*p* < 0.001, Pearson’s coefficient R^2^ = 0.56). These data suggest that owners did not miss significant volumes of urine when collecting urine from their dogs for 24-h collections. Urine creatinine (mg/dl); Urine volume (mls). (PNG 269 kb)

## Abbreviations

ALL: Allantoin
BCS: Body condition score
BW: Body weight
CE: Capillary electrophoresis
HX: Hypoxanthine
LBM: Lean body mass
LUTS: Lower urinary tract signs
MER: Maintenance energy requirement
NRC: National research council
UA: Uric acid
USG: Urine specific gravity
VMTH: Veterinary medical teaching hospital
VUH: Voiding urohydropropulsion
X: Xanthine
