# Supplementary material for: Evaluation of dogs with genetic hyperuricosuria and urate urolithiasis consuming a purine restricted diet: a pilot study
Source: BMC Vet Res. 2017 Feb 8;13:45. doi: 10.1186/s12917-017-0958-y (PMC5299647; doi:10.1186/s12917-017-0958-y)
Supplement: Additional file 1: Table S1. — Average nutrient analysis of the test diet. (DOCX 68 kb) [file 12917_2017_958_MOESM1_ESM.docx]

**Table S1**

Average nutrient analysis of the test diet^a^. For the conversion per 1000 kcal, metabolizable energy (ME) was obtained from the NRC 2006 total dietary fiber prediction equation (National Research Council 2006; ME= 3920 kcal/kg as fed)

| Nutrient | g/100 g as fed | g/1000 kcal |
| --- | --- | --- |
| Moisture | 9 | 23.0 |
| Crude protein | 19.5 | 49.7 |
| Crude fat | 15.0 | 38.3 |
| Crude fiber | 1.0 | 2.6 |
| Total dietary fiber | 6.5 | 16.6 |
| Ash | 6.1 | 15.6 |
| Calcium | 0.82 | 2.09 |
| Phosphorus | 0.63 | 1.61 |
| Sodium | 0.33 | 0.84 |
| Chloride | 0.9 | 2.30 |
| Potassium | 0.84 | 2.14 |
| Magnesium | 0.06 | 0.15 |

**Ingredients:** rice, corn, wheat, dried egg powder, chicken fat, corn gluten meal, natural flavors, monocalcium phosphate, wheat gluten, calcium carbonate, anchovy oil, potassium chloride, salt, fructo-oligo-saccharides, taurine, potassium citrate, L-lysine, choline chloride, borage oil, vitamins. L-carnitine, trace minerals, marigold extract, preserved with natural tocopherols, rosemary extract and citric acid.
