# Supplementary material for: Evaluation of dogs with genetic hyperuricosuria and urate urolithiasis consuming a purine restricted diet: a pilot study
Source: BMC Vet Res. 2017 Feb 8;13:45. doi: 10.1186/s12917-017-0958-y (PMC5299647; doi:10.1186/s12917-017-0958-y)
Supplement: Additional file 2: Table S2. — List of acceptable low purine treats provided to owners. (DOCX 39 kb) [file 12917_2017_958_MOESM2_ESM.docx]

**Table S2**

**List of acceptable low purine treats provided to owners**

**Treat**  mg uric acid/100 g  kcal/100 g

Strawberry  21 32

Raspberry 18 52

Pineapple 19 50

Apple (without skin) 14 48

Pear 12 58

Peach 21 39

Melon cantaloupe 33 34

Cucumber (peeled) 7.3 12

Carrot 17 41

Banana 57 89

Cottage cheese (2%) 9.4 86

Adapted from Brule and others 1988 and from http://www.nal.usda.gov
