# Supplementary material for: Evaluation of dogs with genetic hyperuricosuria and urate urolithiasis consuming a purine restricted diet: a pilot study
Source: BMC Vet Res. 2017 Feb 8;13:45. doi: 10.1186/s12917-017-0958-y (PMC5299647; doi:10.1186/s12917-017-0958-y)
Supplement: Additional file 3: Chart S1. — Owner questionnaire provided at each visit. (DOCX 215 kb) [file 12917_2017_958_MOESM3_ESM.docx]

**Chart S1**

**Owner Questionnaire Provided at Each Visit**

**On a scale of 1-5 please answer the following questions:**

1. **How well does your dog like his or her current diet?**

(1 = very much, 5 = needs significant coaxing)

1 2 3 4 5

1. **How athletic is your dog?**

(1 = very athletic, 5 = goes outside for voiding purposes only)

1 2 3 4 5

1. **How would you rate your dog’s body condition score based on the document provided?**

1 2 3 4 5


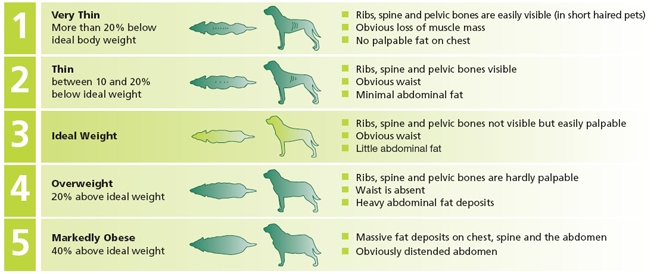


1. **How would you rate your dog’s coat condition?**

(1 = shiny and excellent, 5 = dry and dull)

1 2 3 4 5
