# Supplementary figures and images for: Evaluation of dogs with genetic hyperuricosuria and urate urolithiasis consuming a purine restricted diet: a pilot study
Source: BMC Vet Res. 2017 Feb 8;13:45. doi: 10.1186/s12917-017-0958-y (PMC5299647; doi:10.1186/s12917-017-0958-y)

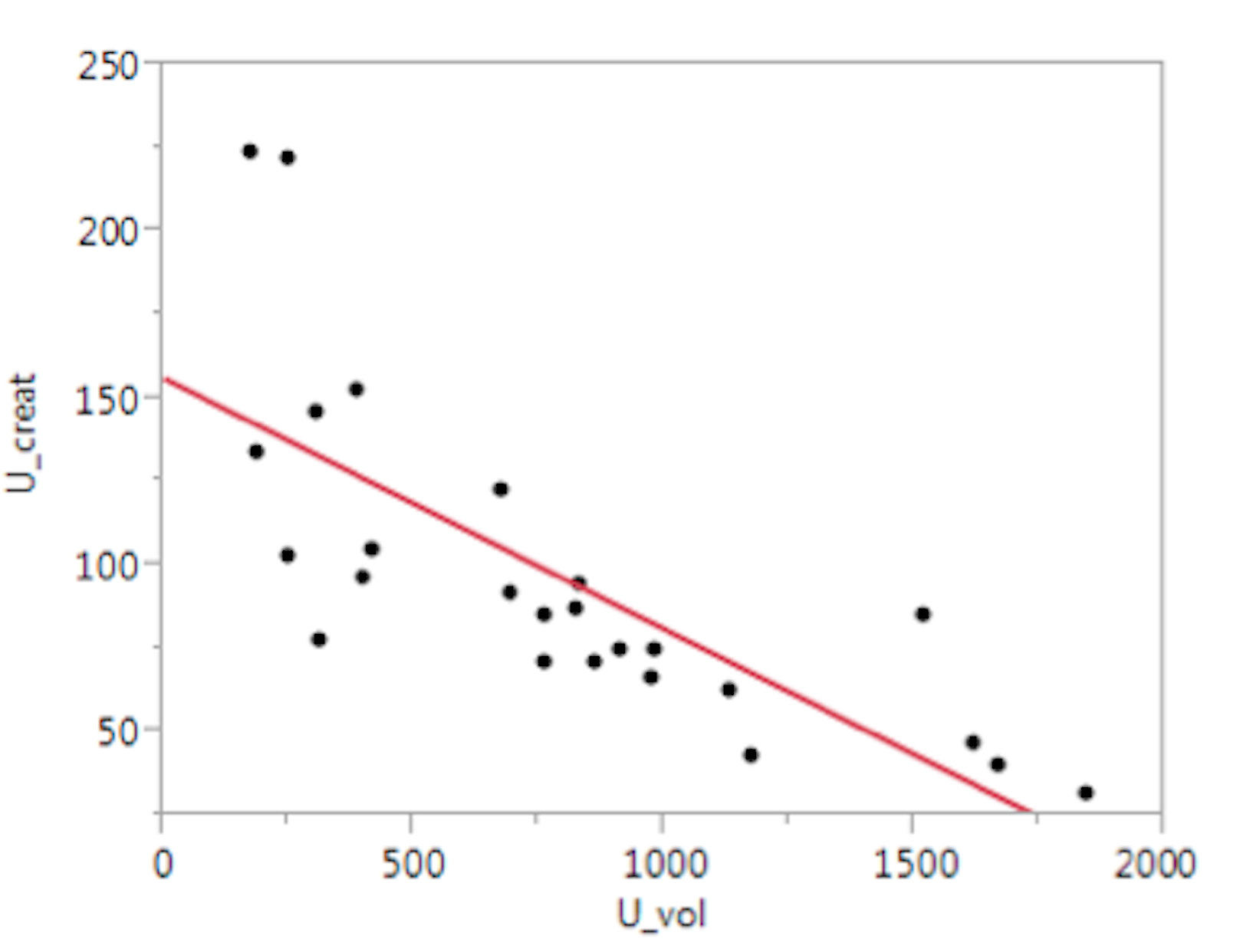

Supplement: Additional file 4: Figure S1. — Evaluation of the correlation between urine volumes to urine creatinine. There was a normal distribution of the residuals, as well as homoscedasticity (indicating homogeneity of the variance and the absence of outliers). The correlation was highly significant and negative (p < 0.001, Pearson’s coefficient R2 = 0.56). These data suggest that owners did not miss significant volumes of urine when collecting urine from their dogs for 24-h collections. Urine creatinine (mg/dl); Urine volume (mls). (PNG 269 kb) [file 12917_2017_958_MOESM4_ESM.png]
